# Rate of occult hepatitis B virus infection among individuals with tuberculosis in northeastern Iran: A molecular epidemiological study

**DOI:** 10.1016/j.jve.2023.100333

**Published:** 2023-06-19

**Authors:** Sanaz Ahmadi Ghezeldasht, Saman Soleimanpour, Mohammad Reza Hedayati-Moghaddam, Moein Farshchian, Seyed Abdolrahim Rezaee, Arman Mosavat

**Affiliations:** a Blood Borne Infections Research Center, Academic Center for Education, Culture, and Research (ACECR), Razavi Khorasan, Mashhad, Iran; b Antimicrobial Resistance Research Center, Bu-Ali Research Institute, Mashhad University of Medical Sciences, Mashhad, Iran; c Department of Microbiology and Virology, School of Medicine, Mashhad University of Medical Sciences, Mashhad, Iran; d Division of Oncology, Laboratory of Cellular Therapy, Department of Medical and Surgical Sciences for Children and Adults, University Hospital of Modena and Reggio Emilia, Modena, Italy; e Immunology Research Center, Inflammation and Inflammatory Diseases Division, Mashhad University of Medical Sciences, Mashhad, Iran

**Keywords:** HBV, Hepatitis B virus, Northeastern Iran, OBI, Occult hepatitis B virus infection, qPCR, Qualitative real-time PCR, TB, Tuberculosis

## Abstract

One third of the world population has a history of exposure to the hepatitis B virus (HBV), and two billion people are infected with latent tuberculosis (TB). Occult hepatitis B infection (OBI) is defined as the presence of replicative-competent HBV DNA in the liver with detectable or undetectable HBV DNA in the serum of individuals testing negative for the HBV surface antigen (HBsAg). Screening with HBV DNA could identify OBI and significantly reduce carriers and complications of chronic hepatitis B (CHB). This study aims to assess HBV serological markers and OBI molecular diagnosis among people with TB in Mashhad, northeastern Iran. We have performed HBV serological markers (HBsAg, HBc antibodies (Ab) and HBs Ab) in 175 participants. Fourteen HBsAg^+^ sera were excluded for further analysis. The presence of HBV DNA (*C*, *S*, and *X* gene regions) was assessed by the qualitative real-time PCR (qPCR) method. Frequencies of HBsAg, HBc, and HBs Ab were 8% (14/175), 36.6% (64/175), and 49.1% (86/175), respectively. Among these 42.9% (69/161) were negative for all HBV serological markers. The *S*, *C*, and *X* gene regions were positive in 10.3% (16/156), 15.4% (24/156), and 22.4% (35/156) of participants, respectively. The total OBI frequency was estimated at 33.3% (52/156) when based on detecting one HBV genomic region. Twenty-two and 30 participants had a seronegative and seropositive OBI, respectively. Thorough screening of high-risk groups with reliable and sensitive molecular methods could lead to OBI identification and decrease CHB long-term complications. Mass immunization remains critical in preventing, reducing, and potentially eliminating HBV complications.

## 1 Introduction

Occult hepatitis B virus infection (OBI) is a challenging clinical entity, presently defined as the presence of replication-competent hepatitis B (HBV) DNA in the liver with detectable or undetectable HBV DNA in serum of subjects with negative hepatitis B surface antigen (HBsAg).^1^, ^2^, ^3^ A definition was provided by J.P. Allain in 2004, who defined OBI as the presence of HBV DNA without HBsAg, with or without the presence of HBV antibodies (Ab) outside the acute phase window period.^4^ The identification of OBI is complex and controversial. OBI can be seen in two situations: seropositive- and seronegative OBI. In seropositive-OBI subjects, HBV DNA can be detected in serum, and anti-HBc/anti-HBs IgG are also positive, or with only positive anti-HBc IgG. Most cases of OBI are clinically asymptomatic. In seronegative-OBI subjects, HBV DNA can only be detected in liver tissue or serum, serum anti-HBc IgG/anti-HBs IgG being also negative.^5^

An International Workshop on OBI in 2008, endorsed by the European Association for the Study of the Liver (EASL),^6^ as well as the Taormina Consensus Conference in 2008, introduced a cut-off value for serum HBV DNA (<200 IU/mL). Hence, cases whose serum HBV DNA levels were comparable to those with overt HBV infection are generally due to infection with HBV escape mutants and should be labelled as “false” OBI.^7^ The OBI serology pattern is as follows; 35% of individuals are anti-HBs Ab and anti-HBc Ab positive, 42% only anti-HBc Ab positive (seropositive-OBI), and 22% are negative for both anti-HBs Ab and anti-HBc Ab indices (seronegative-OBI, and have very low levels of HBV DNA). The HBV DNA detection rate is higher in anti-HBc Ab positive and anti-HBs negative individuals. When an individual has both antibodies, intermediate levels of HBV DNA can be detected.^8^

The cellular and humoral immune response pressure on HBV membrane proteins is one of the main mechanisms in OBI. Masking of HBsAg by the immune complex [HBsAg-anti-HBs Ab] is another hypothesized mechanism. Moreover, simultaneous infection with hepatitis D and C viruses may inhibit HBV proliferation and reduce HBsAg production.^9^

According to the World Health Organization (WHO) report, about one third of the world population have had past or present exposure to HBV infection, and two billion people are infected with latent tuberculosis (TB) worldwide. Although implementing HBsAg testing in routine screening of blood donors in the early 1970s has dramatically improved transfusion safety, HBV transmission remains the most frequent transfusion-transmitted viral infection.^10^, ^11^, ^12^ OBI can be considered a potential risk factor for individuals with thalassemia, hemophilia, and in the context of hemodialysis.^13^ Additionally, OBI is of significance in bone marrow and organ transplantation.^5^^,^^14^^,^^15^

The gold standard for OBI diagnosis is the HBV DNA identification in liver biopsy. However, liver tissue specimens are only rarely available, and standardization and HBV DNA assays in liver tissue have yet to be approved by the US Food and Drug Administration (FDA).^16^ Real-time PCR can generally detect <10 HBV DNA copies. Thus, evaluating several HBV genome regions could increase the OBI detection rate.^17^ Studies have shown that high titers of anti-HBs Ab had a protective effect,^10^^,^^18^ and for this reason, countries such as Germany, Austria, and Japan allow transfusion of units with anti-HBs Ab titer higher than 100 IU/L.^9^^,^^19^

The results of several studies have shown that 0–36% of individuals on hemodialysis and about 10% of those on peritoneal dialysis have OBI. The OBI prevalence in individuals with HIV has been reported at 0–89%.^20^ This wide range indicates the existence of issues and limitations in the diagnosis of OBI. In Iran, the OBI prevalence is two in 50,000 blood donors and 14% in cryptogenic cirrhosis individuals, while the prevalence in seropositive patients is 2.27% and zero in blood donors.^5^ Iran has been classified as a mid-endemic area in HBV prevalence.^21^ In Iran, since 1993, mass HBV immunization has been introduced and implemented.^22^^,^^23^

Mashhad, the capital of Razavi Khorasan, is the second most populated city in Iran, with 3,372,660 inhabitants according to the last census in 2016. In addition, it is a pilgrimage city with a considerable moving population of around 20–30 million per year. It has a highly mixed population of Arab, Turkic, Mongolian, and Afghan tribes (National Population and Housing Census 2016, Iran; https://www.amar.org.ir/english). The OBI prevalence in high-risk groups such as individuals with hepatocellular carcinoma, chronic hepatitis C (HCV), HIV, blood donors, and hemodialysis has been reported. However, there is no information about its prevalence in people with TB. Therefore, this study aims to determine the prevalence of HBV serological indicators (HBsAg, HBc Ab and HBs Ab) and the presence of OBI based on HBV-DNA among people with TB in Mashhad, northeastern Iran.

## 2 Materials and methods

### 2.1 Ethics, study population and settings

The Biomedical Research Ethics Committees of the Academic Center for Education, Culture, and Research (ACECR), Razavi Khorasan, Mashhad, Iran, reviewed and approved this study protocol **[IR.ACECR.JDM.REC.1397.017]**.

Non-probability sampling was performed unrestricted to gender and age from March 2020 to March 2022. In addition, a questionnaire collected demographic information. Written informed consent was obtained from all participants, and for the participation of patients younger than 18 years, consent was obtained from their guardians.

All newly diagnosed individuals with TB by expert infectious disease physicians or pulmonologists at the Referral Pulmonary Diseases and Tuberculosis Ward, Shariati Teaching Hospital, Mashhad University of Medical Sciences, Mashhad, Iran, were enrolled into the study. The diagnosis of TB was defined by respiratory symptoms, sputum smear, and Ziehl-Neelsen (ZN) smear. Exclusion criteria included coinfection with HIV, HCV and autoimmune diseases.

### 2.2 HBV serological assays

Blood samples (3 mL) were collected from 175 participants, and serum tested by the enzyme-linked immunosorbent assay (ELISA) for HBV serological markers (HBsAg, HBc Ab and HBs Ab) using 4th generation ELISA kits (Dia. Pro, Italy). Confirmed HBsAg positive samples were excluded (overt HBV infection).

### 2.3 Qualitative real-time PCR, TaqMan assay (qPCR)

Primer pairs to amplify the fragments of the HBV covering the *pre-core*, *S*, and *X* regions were designed by the AlleleID® software Ver.7.5 (PREMIER Biosoft, USA). To design HBV-specific primers and probes (*pre-core*, *S*, and *X* genes), the complete sequence of the HBV DNA genome (GenBank accession no. NC_003977.2) was obtained from the NCBI database (https://www.ncbi.nlm.nih.gov/nuccore/NC_003977.2/). Primer sequences were sent to Arian Gene Gostar (https://www.ariangene.com) for synthesis by Metabion International AG, Germany (https://www.metabion.com). Amplification of a single product for each primer set was confirmed by 2% agarose gel electrophoresis analysis. The PCR products were then sequenced by Applied Biosystems (Seq Lab, Germany) to confirm primer design validity. The sequences of designed primers and probes are shown in Table 1.

**Table 1:** Sequences of designed primers and probes of hepatitis B (HBV) genes.

HBV genes	Sequence (5'→3′)patitis B	Nucleotide position	Amplicon size (bp)
*Surface* region	Forward-AGGGCATACTACAAACTTTG Reverse-GAGGATGAGTGTTTCTCAAA Probe: FAM-CTCCACCAATCGCCAGTCAG-BHQ1	3061–3080 3168–3149	**108**
*Pre-core* region	Forward-GTACGAGATCTTCTAGATAC Reverse-GTAGCTAGAGTCATTAGTTC Probe: FAM-AAGCCTTAGAGTCTCCTGAGC-BHQ1	1981–2000 2111–2092	**131**
*X* region	Forward-CAAGGTCTTACATAAGAGGA Reverse-CAGTCTTTAAACAAACAGTC Probe: FAM-CAATGTCAACGACCGACCTTG-BHQ1	1645–1664 1734–1715	**90**

The qualitative real-time PCR (qPCR) assay was performed by a CFX96 (Bio-Rad, USA) PCR machine using Premix Ex Taq (Probe qPCR) (TaKaRa, Cat. No. RR390Q, Japan) on DNA extracted from serum samples using the QIAamp® DSP Virus Kit (QIAGEN, Germany) according to the manufacturer's instructions. Positive control of the HBV commercial kit (artus HBV QS-RGQ kit, QIAGEN, Germany) was used to control qPCR experiments.

All reactions were optimized in a final volume of 20 μL (qPCR master mix: 10 μL; forward primer: 1 μL; reverse primer: 1 μL; probe: 0.3 μL; DNA template: 7.7 μL). The temperature-time profile of qPCR for *S*/*X* genes was as follows; 95 °C/30 s, 95 °C/5 s and 54 °C/30 s (40 cycles). Similarly, the *C* gene was detected according to the previous protocol with annealing at 55 °C/30 s.

### 2.4 Statistical analysis

Statistical analysis was performed using SPSS software V 13.0 (SPSS, Chicago, IL). The normality of the data for each variable was checked before data analysis. The chi-square or Fisher's exact test was used for qualitative variables. The *p*-value was considered statistically significant if *p* < 0.05.

## 3 Results

### 3.1 HBV serological markers

The present study enrolled 165 TB patients (pulmonary and extrapulmonary). 36.6% (64/175) of patients had HBc Ab, which indicated a resolved infection or history of exposure to HBV.

The overall rate of anti-HBs Ab among these subjects was 49.1% (86/175). In other words, they were immune or not susceptible to HBV infection. On the other hand, the presence of HBsAg indicates that the subjects are infectious, which occurred in 8% (14/175) who had overt HBV infection and were therefore excluded from further analysis.

Moreover, 42.9% (69/161) of individuals were negative for both HBc Ab/HBs Ab, indicating non-immune or susceptibility to HBV infection. HBc Ab and HBs Ab in HBsAg negative individuals are shown in Table 2.

**Table 2:** Numbers of HBc Ab and HBs Ab markers in HBsAg^−^ individuals.

Serum marker	No.	(%)
Both HBcAb/HBsAb seronegative	69	42.9%
Only HBcAb seropositive	11	6.8
Only HBsAb seropositive	40	24.8
Both HBcAb/HBsAb seropositive	41	25.5
Total	161	100

### 3.2 OBI definition and frequencies of *S, C, and X* gene regions in individuals with TB

The presence of one HBV genomic region (*S/C*/*X* genes) was considered in this study to be consistent with OBI. Seronegative- and seropositive-OBI were defined based on HBV serological markers in individuals with TB.^2^^,^^3^^,^^6^ The limit of detection (LOD) was determined according to each HBV gene threshold (Ct/Cq value). The threshold of ≤38 was considered positive. The Ct values of detected HBV genes (*S/C*/*X* genes) are shown in Table 3.

**Table 3:** Ct values of *S/C*/*X* HBV genomic regions in subjects with tuberculosis.

No.	Gene names	Ct values (Log_8_)	Ct values of HBV positive control (Log_8_)
**1**	*S* region	34.9	18.5
**2**		36.8	
**3**		34.6	
**4**		35.8	
**5**		35.3	
**6**		36.2	
**7**		35.5	
**8**		20	
**9**		33.9	
**10**		35.6	
**11**		36.9	
**12**		35.3	
**13**		34.1	
**14**		36.05	
**15**		17.7	
**16**		26.4	
**1**		31.3	
**2**	*C* region	33.5	16.1
**3**		34.4	
**4**		25.3	
**5**		33.5	
**6**		33.7	
**7**		34.6	
**8**		17.8	
**9**		32.5	
**10**		34.2	
**11**		34.2	
**12**		33.8	
**13**		29.2	
**14**		33.2	
**15**		28.7	
**16**		30.5	
**17**		17.4	
**18**		24.3	
**19**		30.5	
**20**		32.8	
**21**		34.2	
**22**		30.2	
**23**		28.2	
**24**		34.2	
**1**	*X* region	36.7	17.5
**2**		34.6	
**3**		36.4	
**4**		36.1	
**5**		25.4	
**6**		34.1	
**7**		35.9	
**8**		37.3	
**9**		17.8	
**10**		34	
**11**		36.3	
**12**		35.6	
**13**		35.7	
**14**		36.7	
**15**		35.2	
**16**		14.4	
**17**		35.2	
**18**		36.5	
**19**		24.4	
**20**		32.9	
**21**		34.4	
**22**		36.7	
**23**		35.7	
**24**		32.6	
**25**		30.3	
**26**		36.7	
**27**		35.4	
**28**		31.2	
**29**		36.2	
**30**		31.1	
**31**		36.5	
**32**		33.3	
**33**		37.1	
**34**		37.3	
**35**		30.4	

Five serum samples had evaporated during long-term preservation at −70 °C (161–5 = 156 sera remained for further analysis). The HBV DNA genomic region was detected in 33.3% (52/156) of people with TB. Of these, 12.2% (19/156) were positive for two genomic regions. Frequencies of the *S,* C, and *X* regions were 16/156 (10.3%), 24/156 (15.4%) and 35/156 (22.4%), respectively. The *C* and *S* genomic regions were detected simultaneously in 2.6% (4/156) of HBsAg negative individuals.

The *X* and *S* regions were detected in 1.9% (3/156) of HBsAg negative individuals. The highest simultaneous presence of two HBV genomic regions involved the *X* and *C* genomic regions (7.7% or 12 cases).

### 3.3 Frequency of OBI based on genomic regions and HBV antibodies

Among individuals who were positive for at least one HBV genomic region (52/156), 32 (30.8%) were negative in terms of HBc Ab (seronegative-OBI). There were no significant differences in the frequencies of OBI according to HBc Ab.

Out of 19 individuals with two HBV genomic regions detected (qPCR positive for two genes), 12 were HBc Ab negative, and seven positive (Table 4).

**Table 4:** Frequencies of occult hepatitis B infection based on qualitative qPCR and HBc Ab/HBs Ab serostatus.

HBc Ab	qPCR-positive of one genomic region		
	Negative	Positive	*p*-value
Negative *n* (% within HBcAb)	72 (69.2%)	32 (30.8%)	0.337
Positive *n* (% within HBcAb)	32 (61.5%)	20 (38.5%)	
**HBcAb**	**qPCR-positive of two genomic regions**		
	**Negative**	**Positive**	***p*-value**
Negative *n* (% within HBcAb)	92 (88.5%)	12 (11.5%)	0.729
Positive *n* (% within HBcAb)	45 (86.5%)	7 (13.5%)	
**HBcAb/HBsAb**	**qPCR-positive of one genomic region**		
	Negative	Positive	***p*-value**
Both HBc Ab/HBs Ab seronegative *n* (% HBV serostatus)	42 (65.6%)	22 (34.4%)	0.531
Only HBc Ab seropositive *n* (% HBV serostatus)	6 (54.5%)	5 (45.5%)	
Only HBs Ab seropositive *n* (% HBV serostatus)	30 (75%)	10 (25%)	
Both HBc Ab/HBs Ab seropositive *n* (% HBV serostatus)	26 (63.4%)	15 (36.6%)	
**HBcAb/HBsAb**	**qPCR-positive of two genomic regions**		
	Negative	Positive	***p*-value**
Both HBc Ab/HBs Ab seronegative *n* (% HBV serostatus)	55 (85.9%)	9 (14.1%)	0.715
Only HBc Ab seropositive *n* (% HBV serostatus)	10 (90.9%)	1 (9.1%)	
Only HBs Ab seropositive *n* (% HBV serostatus)	37 (92.5%)	3 (7.5%)	
Both HBc Ab/HBs Ab seropositive *n* (% HBV serostatus)	35 (85.4%)	6 (14.6%)	

Frequencies of HBc Ab/HBs Ab in OBI subjects were 28.8% (15/52) and 31.5% (6/19) with one and two qPCR-positive HBV genomic regions, respectively, with no significant differences among groups for the presence of HBV antibodies (Table 4).

## 4 Discussion

Coinfection with HBV in people with TB increases the risk of treatment failure,^24^ can reactivate latent TB,^25^^,^^26^ and induce drug-related liver injury.^27^, ^28^, ^29^ Some authors have recommended that HBV screening in people with TB could improve its management due to the possibility of coexistence with HBV in endemic areas.^30^ The hepatotoxicity of anti-TB drugs increases the risk of hepatitis, leading to treatment interruption. A common adverse drug reaction, with anti-TB drugs, eg drug-induced liver injury (DILI), in chronic liver disease, is challenging because it is more common within this population.^31^ Contradictory results have been obtained from epidemiological studies on the impact of HBV infection on the incidence and severity of TB drug DILI.^27^^,^^32^ Available data on HBV incidence, especially OBI in people with TB, is rare.^33^ Therefore, identifying OBI should be clinically significant in people with TB.

In the present study, the overall prevalence of OBI among individuals with TB. was 33.3% (52/156), when taking into consideration the presence of only one HBV genomic region. In addition, 61.5% (32/52 cases) of OBI/TB individuals were HBc Ab negative (seronegative-OBI), while 38.5% (20/52 cases) were seropositive (seropositive-OBI). More than half of these had both HBc and HBs antibodies.

Previous studies have reported HBV infection prevalence in individuals with TB: Rio de Janeiro, Brazil (26.8%; 95% CI: 19.7–31.9),^34^ Argentina (19.8%; 95% CI: 14.3–26.2),^35^ Taiwan (11.7%; 95% CI: 6.8–15.5)^36^ and Georgia (13%; 95% CI: 9.5–17.5).^37^

Overall, the prevalence of OBI in different world regions varies from 1 to 87%. It is affected by several factors, such as HBV prevalence, study population, HBV immunization programs, and the sensitivity of diagnostic assays.^38^ However, OBI has also been reported in geographic areas with low HBV endemicity. The OBI prevalence among the general population in China is 45.5% with genotype B,^39^ and in South Korea, 1.7–6% with genotype C2.^40^ In Taiwan, 10.9% of vaccinated children and 0.11% of blood donors had OBI.^41^ In Egypt, it ranges from 1.4 to 26.8%.^42^ A low OBI prevalence among blood donors in Beirut, Lebanon, has also been reported (4.6%).^43^ In Turkish HIV-infected individuals, the prevalence was 1.4%.^44^

Various rates of OBI have been reported from different regions of Iran, from zero^45^ to 63% among the HIV-positive population in the Fars province.^46^

In two systematic reviews of studies conducted over 25 years (1990–2014), the overall rate of HBV infection among the general population in Iran was estimated at 2.2%–3%, ranging from 0.7% to 0.9% in the Kermanshah and Kurdistan provinces, respectively, to 8.9% in the Golestan province.^47^^,^^48^ Therefore, Iran could be considered a country with a low/intermediate level of HBV infection.

A study has shown a prevalence of OBI in two of 50,000 blood donors.^49^ Kalantari et al. have shown 0% in hemodialysis patients,^50^ and Hashemi et al. 14% in cryptogenic liver cirrhosis patients.^51^ In a study conducted by Ramezani et al. among 289 Iranian hemodialysis patients, 18 had isolated HBc Ab (6.2%), with HBV-DNA detectable in 50% of them (9/18).^52^ In another study among HBc positive individuals in Zahedan County, OBI was at 2.27%.^53^

This wide range of results stresses the issues with the diagnosis of OBI which needs identifying the target population for screening. Adequate and accurate OBI diagnosis is vital in people with hepatitis C, HIV, CHD and in those on hemodialysis, as well as transplant recipients. In 2012, Mahmoud et al. conducted a study on 100 HBsAg negative blood samples from Sudanese blood donors among whom 42% were positive for HBc Ab and 8% for HBe Ab; The SYBR Green Real-Time PCR method was implemented for three different HBV genomic regions (*S*, *C* and *X*). The *C* region gene was identified in 90.5% (38/42), and *X* and *S* regions in 71.4% and 42.8%, respectively.

In the current study, the frequency of *S*, *C*, and *X* genomic regions were 10.3%, 15.4%, and 22.4%, respectively, with the highest frequency observed in the *X* genomic region. The incidence of OBI (one observed genomic region) in HBc Ab seronegative and seropositive participants was 30.8% and 38.5%, respectively. HBc Ab screening can reduce the risk of HBV transmission but may also provide false-positive results.^54^

In 2016 the WHO announced that viral hepatitis should be eliminated by 2030; however, the difficulty in identifying and treating individuals with OBI may prevent achieving this goal. In 2008, the Taormina Expert Meeting defined OBI as the presence of replicative-competent HBV DNA in the liver of individuals negative for HBsAg testing with the amount of serum HBV-DNA usually lower than 200 IU/mL or undetectable.^6^

Low levels of HBV replication may be associated with OBI. Compared to HBsAg^+^ infection, OBI has lower (<200 IU/mL) or undetectable HBVDNA in serum. The molecular basis of the occult infection is closely related to HBV genomes in the form of episomal covalently closed circular DNA (cccDNA) and a low state of replication. The detection of HBV DNA in serum/plasma is intermittent or at a low viremic range.^55^ The cccDNA in OBI cases is an entirely appropriate replicate.^56^ In very few cases, OBI has been ascribed to mutations influencing viral replication.^57^ The low level of transcriptionally active cccDNA in hepatocytes results in low HBV transcripts and subsequent proteins, with undetectable HBsAg. This suggests that the host rather than viral factors are more critical in determining OBI.

The present study has some limitations; firstly, a larger sample size with relevant clinical data would provide more reliable results. Secondly, the sensitivity and specificity of the homemade assay should be validated and standardized. Thirdly, when considering the complexity of OBI and our findings compared to other authors, our results should be interpreted with caution.

In conclusion, health policy decision-makers should consider incorporating screening for OBI during TB treatment. In addition, improved diagnosis of OBI is necessary for a better comprehension of its molecular epidemiology and pathogenesis, especially in people with TB. Development of more sensitive, standardized, reliable, and validated commercially available assays for OBI diagnosis is needed. Molecular epidemiological-based surveys in high-risk groups worldwide could herald new strategies for reducing HBV complications.

## Funding

This study was supported financially by 10.13039/501100005013the Vice-Chancellor for Research and Technology, Academic Center for Education, Culture, and Research (ACECR), Razavi Khorasan, Mashhad, Iran, under Grant **[ACECR 3055–20, recipient: A. Mosavat]**.

## Authors’ contributions

Performing the experiments and manuscript drafting: SAG and AM; Research advisors: SS, MF, MRH, and SAR; Data analysis and preparing facilities and kits: MRH; Research director, planned, conception and design of the study, revision and finalization of the manuscript: AM. All authors have read and approved the final manuscript.

## Availability of data and materials

All data supporting this study's findings is included into the manuscript and available from the corresponding author upon request.

## Ethics approval/Consent for participation

This study was performed in line with the principles of the Declaration of Helsinki. The study protocol was reviewed and approved by the Biomedical Research Ethics Committee of the Academic Center for Education, Culture, and Research (ACECR), Razavi Khorasan, Mashhad, Iran **[IR.ACECR.JDM.REC.1397.017]**. Written informed consent was obtained and signed by all the participants. All the methods were performed following the relevant guidelines and regulations.

## Consent for publication

Not applicable.

## Declaration of competing interest

The authors declare that they have no known competing financial interests or personal relationships that could have appeared to influence the work reported in this paper.

## Data Availability

Data will be made available on request.
